# Ginsenoside Rd Protects SH-SY5Y Cells against 1-Methyl-4-phenylpyridinium Induced Injury

**DOI:** 10.3390/ijms160714395

**Published:** 2015-06-24

**Authors:** Yang Liu, Ren-Yu Zhang, Jun Zhao, Zheng Dong, Dong-Yun Feng, Rui Wu, Ming Shi, Gang Zhao

**Affiliations:** 1 Department of Neurology, Xijing Hospital, Fourth Military Medical University, Xi’an 710032, China; E-Mails: adrainyoung@126.com (Y.L.); fengdy@fmmu.edu.cn (D.-Y.F.); wurui1124@126.com (R.W.); 2 153rd Hospital of People’s Liberation Army, Zhengzhou 450007, China; 3 Cadet Brigade of Fourth Military Medical University, Xi’an 710032, China; E-Mails: firstzry@126.com (R.-Y.Z.); qiushui1994@hotmail.com (Z.D.); 4 316th Hospital of People’s Liberation Army, Beijing 100091, China; E-Mail: zhaozhi1619@163.com

**Keywords:** ginsenoside Rd, MPP^+^, SH-SY5Y, reactive oxygen species, neuroprotection, Parkinson’s disease

## Abstract

Ginsenoside Rd (GSRd), one of the main active monomer compounds from the medical plant *Panax* *ginseng*, has been shown to promote neuronal survival in models of ischemic cerebral damage. As an extending study, here we examined whether GSRd could exert a beneficial effect in an experimental Parkinson disease (PD) model *in vitro*, in which SH-SY5Y cells were injured by 1-methyl-4-phenylpyridinium (MPP^+^), an active metabolic product of the classical Parkinsonian toxin1-methyl-4-phenyl-1,2,3,6-tetrahydropyridine (MPTP). Our results, from the addition of different concentrations of GSRd (1, 10 and 50 μM), showed that GSRd at 1 and 10 μM could significantly attenuate MPP^+^-induced cell death. This protective effect may be ascribed to its ability to reduce intracellular reactive oxygen species levels, enhance antioxidant enzymatic activities, preserve the activity of respiratory complex I, stabilize the mitochondrial membrane potential and increase intracellular ATP levels. Additionally, the PI3K/Akt survival-signaling pathway was also involved in the protective effect of GSRd. Finally, using a mouse PD model *in vivo*, we also found that GSRd obviously reversed the loss of tyrosine hydroxylase-positive cells in substanitia nigra induced by MPTP. Thus, our findings demonstrated that GSRd showed a significant neuro-protective effect against experimental PD models, which may involve its antioxidant effects and mitochondrial function preservation.

## 1. Introduction

Parkinson’s disease (PD) is the second most common neurodegenerative disorder in the aging population. The characteristic pathological change of PD is the loss of dopaminergic (DA) neurons in substantia nigra (SN) pars compacta. Although the underlying mechanism that induces DA neurons death is still obscure, mitochondrial dysfunction, oxidative stress, reticulum stress, and glial related inflammation are widely accepted to be among the common pathogenesis [1,2,3,4]. Because of their high activity in dopamine metabolism and abundance in lipid component and iron, DA neurons are especially susceptible to these events. Reactive oxygen species (ROS), mainly generated from abnormal mitochondria, disrupt antioxidant defense system, further deteriorate mitochondrial dysfunction and energy insufficiency, and eventually lead to neuronal cell death [5].

Ginsenosides have been proven to be main active components in ginseng, a tonic that has been widely used in traditional Chinese medicine. Among more than 30 ginsenosides that have been isolated, GSRd is one of the major active monomer compounds. In several studies, GSRd has been shown to attenuate neuronal damage. *In vitro* studies, GSRd could relieve cell death caused by oxygen-glucose deprivation [6,7]. *In vivo* studies, GSRd could alleviate ischemic cerebral damage both in histological and functional outcome by providing mitochondrial protection and anti-inflammatory effect [8,9,10]. Additionally, the safety of GSRd has been assessed in stroke patients in clinical investigations [11]. These aforementioned results suggest that GSRd may protect against acute neuronal injuries. However, it is unknown whether GSRd could exert protective effect on neurodegenerative diseases, such as PD. In the present study, we investigated the effects of GSRd on an experimental PD model *in vitro*, in which human neuroblastoma SH-SY5Y cells, a commonly used neuronal cell line, injured by a classical Parkinsonian toxin1-methyl-4-phenylpyridinium (MPP^+^), and a mouse PD model *in vivo*, in which mice were received with 1-methyl-4-phenyl-1,2,3,6-tetrahydropyridine (MPTP).

## 2. Results

### 2.1. GSRd Attenuates MPP^+^-Induced Cell Death

**Figure 1:**
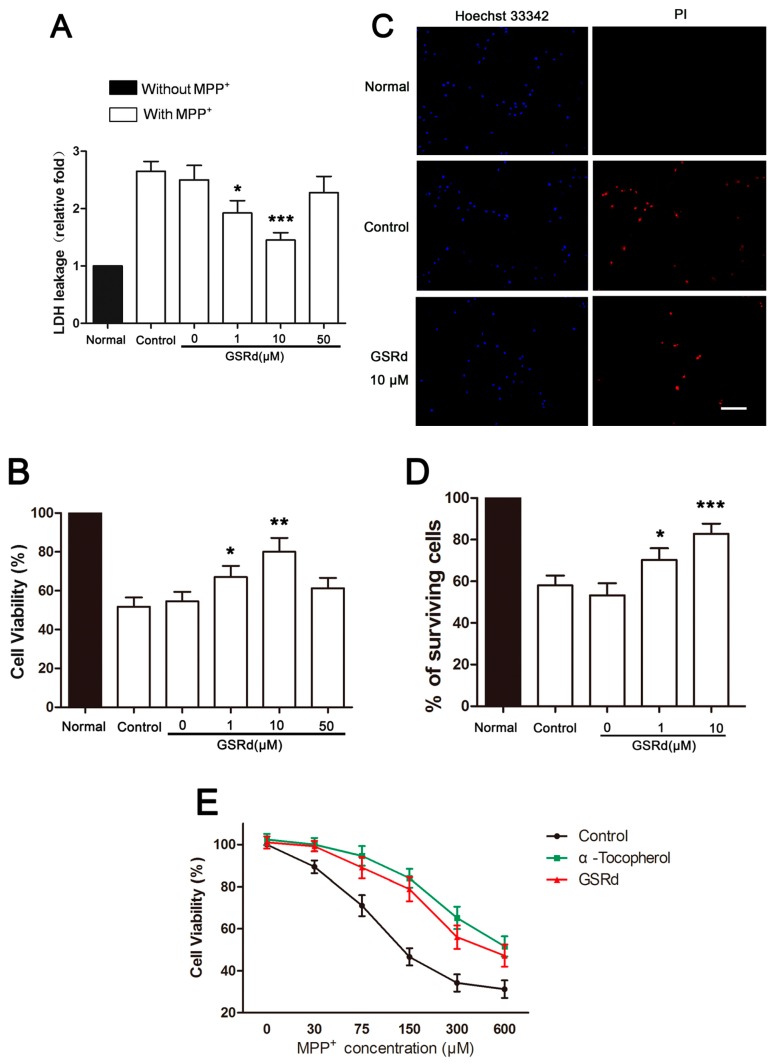
Effects of ginsenoside Rd (GSRd) on viability of 1-methyl-4-phenylpyridinium (MPP^+^) treated SH-SY5Y cells. (**A**,**B**) After incubation in 150 μM MPP^+^ with or without GSRd for 72 h, cell death and cell viability were assessed by lactate dehydrogenase (LDH) leakage and methylthiazolyldiphenyl-tetrazolium bromide (MTT) assays. Control group was treated with MPP^+^ alone, and GSRd 0 μM group was treated with MPP^+^ and vehicle; (**C**) Representative photomicrographs showing Hoechst 33342 and propidium iodide (PI) double stained SH-SY5Y cells of normal, control and GSRd 10 μM group; (**D**) The percentages of surviving cells in different groups based on Hoechst 33342/PI double staining. Scale bar: 50 μm (200×); and (**E**) The protective effect of GSRd at different concentrations of MPP^+^. * *p* < 0.05; ** *p* < 0.01; and *** *p* < 0.001 *vs.* control group.

To test the effect of GSRd on MPP**^+^**-induced cell injury, lactate dehydrogenase (LDH) leakage and methylthiazolyldiphenyl-tetrazolium bromide (MTT) assays were performed. After treatment with 150 μM MPP^+^ for 72 h, cell viability indicated by MTT assay was reduced by >40%, and LDH leakage was about 2.7-fold of the normal. GSRd at 1 μM and 10μM significantly reduced the LDH leakage to 2.1- and 1.3-folds, and attenuated MPP**^+^**-induced cell death to 33.9% and 21.2%, respectively (Figure 1A,B). However, 50 μM GSRd seemingly did not show an obvious protective effect. From these results, we chose 1 and 10 μM in the following studies.

To further examine the protective effect of GSRd, we performed propidium iodide (PI) staining to evaluate MPP**^+^**-induced cell death. Compared with the normal, the number of PI positive cells was increased by addition of MPP**^+^**. Pretreatment of 1 and 10 μM GSRd decreased MPP^+^-induced cell death, and increased percentages of survival cells from 57.3% to 71.1% and 83.6%, respectively (Figure 1C,D).

Other antioxidants, including α-tocopherol, have been shown to attenuate MPP^+^-induced neurotoxicity [12]. To assess whether GSRd exerts direct protective effect on MPP^+^ insult or just inert MPP^+^, we incubated SH-SY5Y cells with different concentrations of MPP^+^ in combination with constant amount of GSRd or α-tocopherol as the positive control. The viability of SH-SY5Y cells decreased obviously with the increase of MPP^+^ concentration, as shown by MTT assay (Figure 1E). The addition of α-tocopherol (10 μM) ameliorated cell death caused by different concentrations of MPP^+^. Like α-tocopherol, GSRd (10 μM) showed similar protective effect, even when a relatively high amount of MPP^+^ was present (Figure 1E). This suggested that GSRd may provide direct protection to MPP^+^-induced cytotoxicity, beyong the MPP^+^ neutralizing effects.

### 2.2. GSRd Reduces MPP^+^-Induced Oxidative Stress

**Figure 2:**
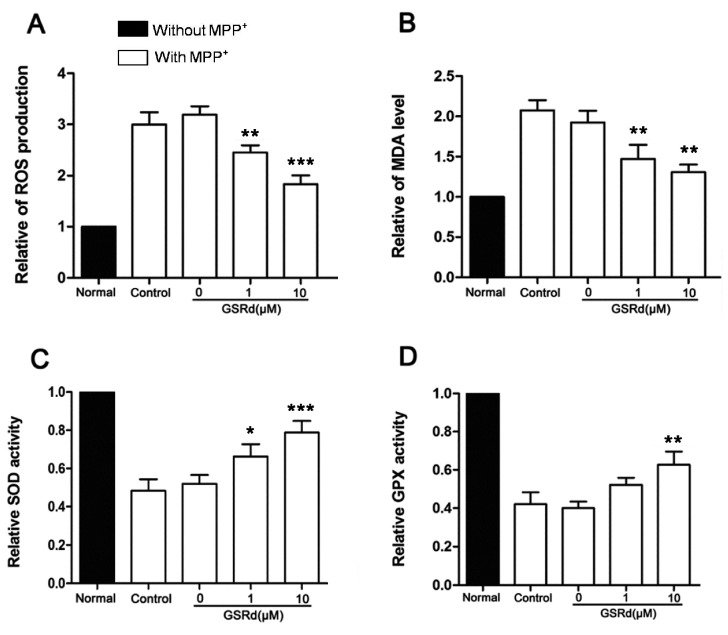
Effects of ginsenoside Rd (GSRd) on reactive oxygen species (ROS), malondialdehyde (MDA) levels and antioxidant enzymatic activities in MPP^+^ treated SH-SY5Y cells. (**A**,**B**) Relative intracellular ROS accumulation and MDA level in 1-methyl-4-phenylpyridinium (MPP^+^) treated SH-SY5Y cells with or without GSRd treatment; (**C**,**D**) Antioxidant enzymatic activities (C,superoxide dismutase, SOD; D, glutathione peroxidase, GPX) in MPP^+^ treated SH-SY5Y cells with or without GSRd. * *p* < 0.05; ** *p* < 0.01; and *** *p* < 0.001 *vs.* control group.

MPP^+^ treatment caused a 3.2-fold increase in intracellular ROS and 2.1-fold increase in malondialdehyde (MDA) amounts. However, GSRd (1 and 10 μM) significantly decreased both levels of ROS and MDA, compared with MPP^+^-treated cells (Figure 2A,B). In addition, MPP^+^ induced decrease in activities of intracellular superoxide dismutase (SOD) and glutathione peroxidase (GPX), two important antioxidant enzymes. However, GSRd could maintain SOD and GPX activities noticeably in the context that it did not affect enzyme activities in normal SH-SY5Y cells (Figure 2C,D).

### 2.3. GSRd Preserves Mitochondrial Function and Inhibits MPP^+^ Induced ATP Depletion

**Figure 3:**
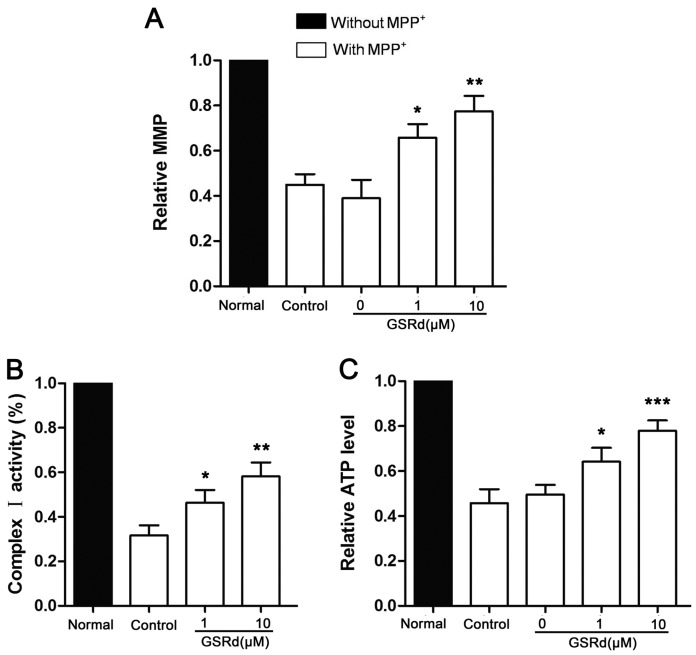
Effects of ginsenoside Rd on mitochondrial function in 1-methyl-4-phenylpyridinium (MPP^+^) treated SH-SY5Y cells. (**A**) Relative changes of mitochondrial membrane potential (MMP); (**B**) relative activity of mitochondrial respiratory complex I; and (**C**) relative intracellular ATP levels. * *p* < 0.05; ** *p* < 0.01; and *** *p* < 0.001 *vs.* control group.

MPP^+^ is a specific antagonist against mitochondrial respiratory complex I, so we investigated the effect of GSRd on mitochondrial depolarization. After MPP^+^ treatment, the fluorescence intensity of Rh 123, a fluorescent dye for detection of mitochondrial membrane potential (MMP) was greatly reduced. However, GSRd (1 and 10 μM) significantly alleviated MPP^+^ induced decrease in MMP from 45.5% to 66.3% and 78.6%, respectively (Figure 3A). Since mitochondrial respiratory complex I is inhibited directly by MPP^+^, further we measured its activity in MPP^+^ treated SH-SY5Y cells with or without GSRd treatment. MPP^+^ insult significantly inhibited complex I, however GSRd administration (1 and 10 μM) induced certain recovery of its activity (Figure 3B).

Also, GSRd treatment (1 and 10 μM) obviously attenuated the decrease of ATP levels induced by MPP^+^ (Figure 3C).

### 2.4. GSRd Prevents Bax Up-Regulation and Modulates Bax/Bcl-2 Ratio

**Figure 4:**
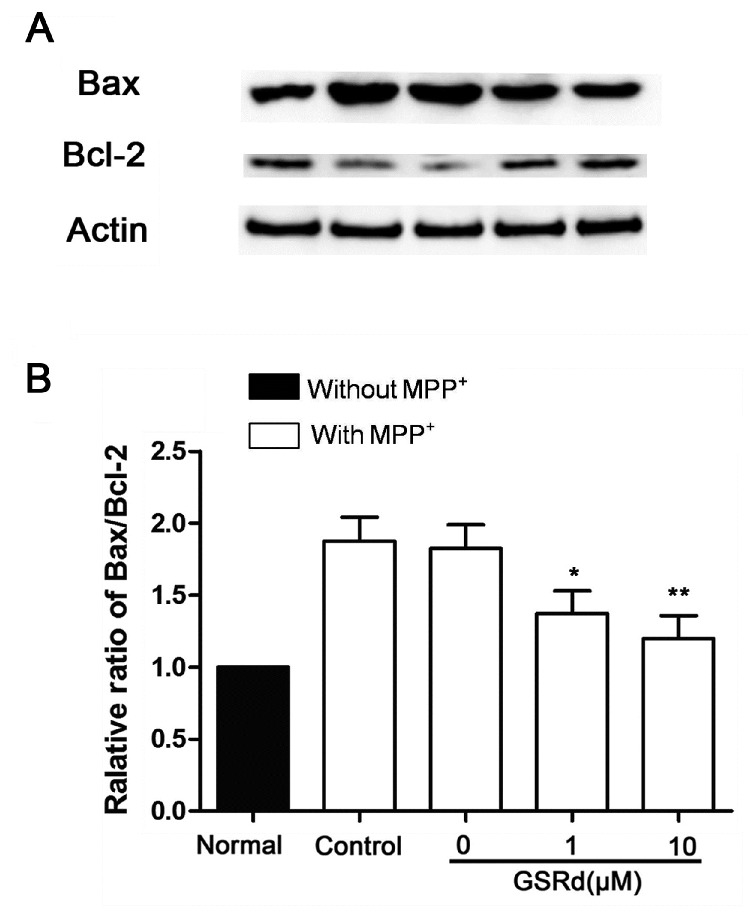
Effects of ginsenoside Rd (GSRd) on Bax expression and Bax/Bcl-2 ratio in 1-methyl-4-phenylpyridinium (MPP^+^) treated SH-SY5Y cells. (**A**) Bax and Bcl-2 expression were determined by Western blot; and (**B**) Bax/Bcl-2 ratio was presented as folds relative to normal. * *p* < 0.05; and ** *p* < 0.01 *vs.* control group.

In cells treated with MPP^+^ alone, the expression of pro-apoptotic Bax increased obviously, and so did the Bax/Bcl-2 ratio. In contrast, administration of GSRd (1 and 10 μM) induced a marked reduction in both Bax expression and Bax/Bcl-2 ratio (Figure 4).

### 2.5. GSRd Prevents p-Akt down-Regulation Induced by MPP^+^ Treatment

**Figure 5:**
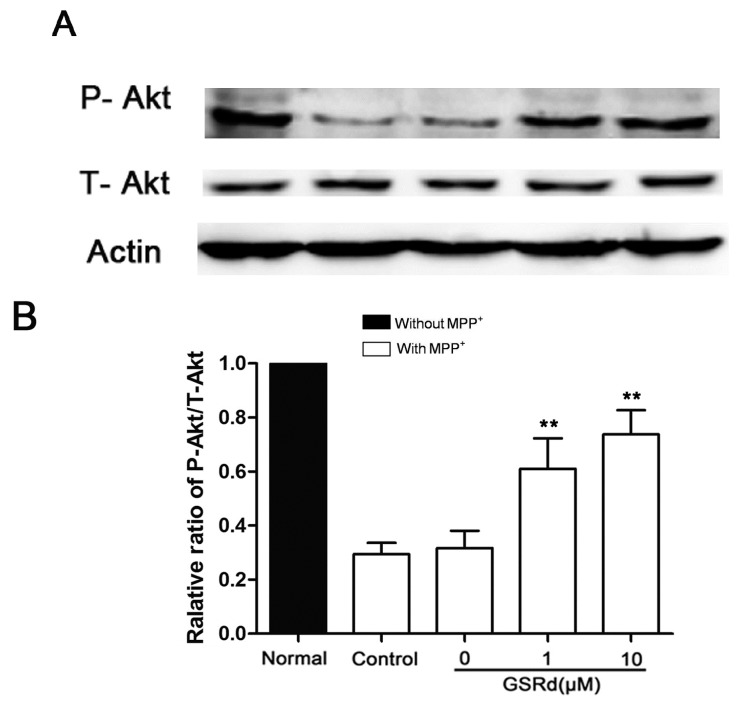

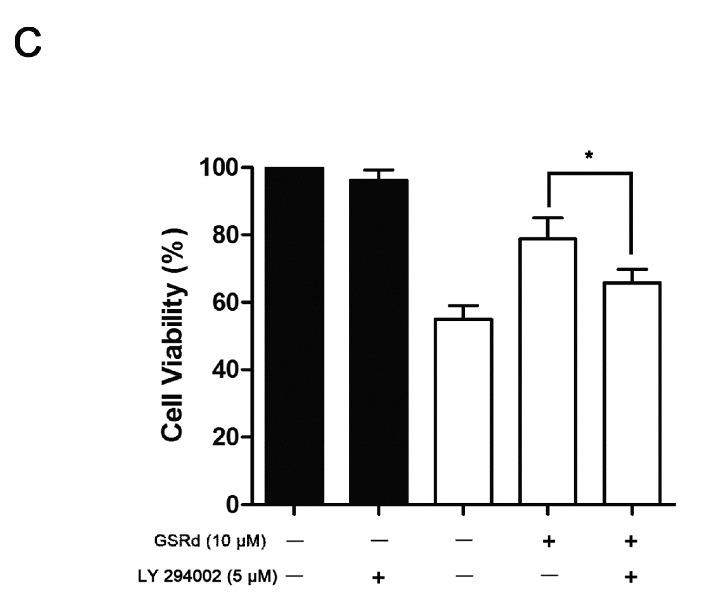
Effects of ginsenoside Rd (GSRd) on Akt phosphorylation in 1-methyl-4-phenylpyridinium (MPP^+^) treated SH-SY5Y cells. (**A**) phosphorylation-Akt (P-Akt) and total-Akt (T-Akt) levels were determined by Western blotting; (**B**) P-Akt/T-Akt ratio was presented as folds relative to normal; and (**C**) administration of LY294002 for 12 h prior to GSRd partly blocked the cytoprotective role of GSRd (cell viability was measured by MTT assay). * *p* < 0.05; and ** *p* < 0.01 *vs.* control group.

In SH-SY5Ycells treated with MPP^+^ alone, phosphorylation-Akt (P-Akt) level was decreased significantly. However, GSRd treatment (1 and 10 μM) could attenuate the down-regulation of P-Akt caused by MPP^+^ (Figure 5B), in keeping with our previous study [13]. This result provided clues that phosphatidylinositol 3-kinase (PI3K)/Akt signaling pathway may be involved in the cytoprotective roles of GSRd in cell death caused by MPP^+^. To further verify this hypothesis, LY294002, a PI3K/Akt pathway inhibitor, was added to SH-SY5Y cells 12 h prior to GSRd. MTT assay showed that LY294002 (5 μM) pre-treatment could partly block the cytoprotective function of GSRd in MPP^+^-insult model (Figure 5C).

### 2.6. GSRd Promotes Survival of TH^+^ Neurons of SN in Vivo

**Figure 6:**
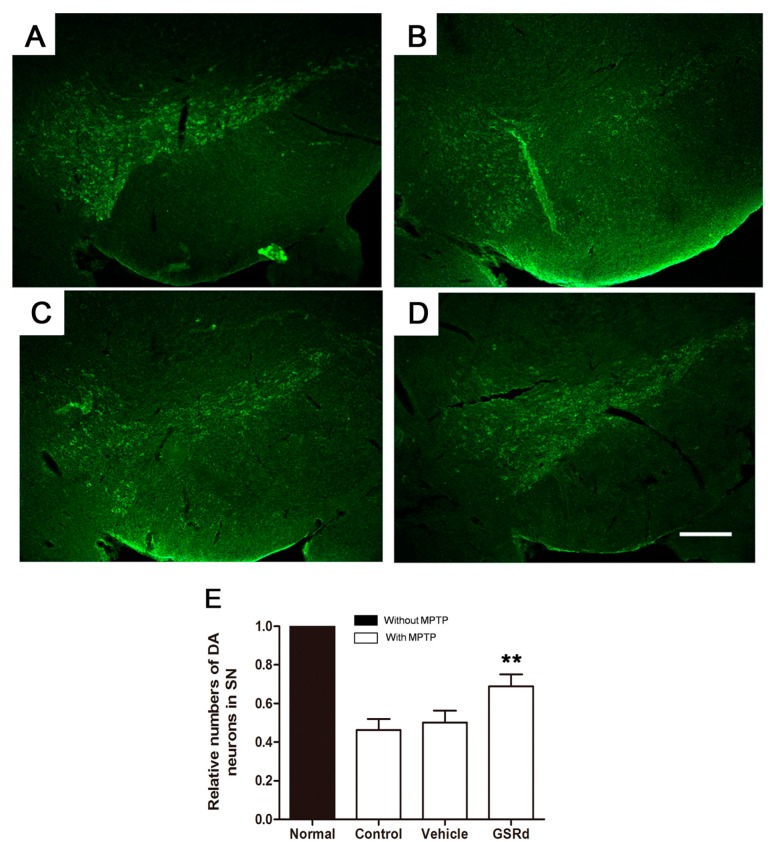
Effects of ginsenoside Rd (GSRd) on the numbers of tyrosine hydroxylase (TH) positive dopaminergic (DA) neurons in 1-methyl-4-phenyl-1,2,3,6-tetrahydropyridine (MPTP) induced Parkinson disease model in C57BL/6J mice. (**A**–**D**) Representative photomicrographs of TH^+^ neurons in substantia nigra (SN); (**E**) The numbers of TH^+^ neurons relative to normal in each group. Scale bar: 100 μm (100×). ** *p* < 0.01 *vs.* control group.

In C57BL/6J mice, MPTP administration resulted in a significant reduction of tyrosine hydroxylase (TH) positive dopaminergic neurons in SN (Figure 6A–D). GSRd treatment (10 mg/kg) prior to MPTP showed protection of TH^+^ cells compared to MPTP and vehicle group (Figure 6E).

## 3. Discussion

After the damage effect of MPTP to human DA neurons was noticed in 1980s, the Parkinsonian symptoms after MPTP intoxication had been remarkably confirmed to resemble those observed in sporadic PD in various animal models. Lipophilic MPTP crosses the blood–brain barrier and is metabolized to toxic MPP^+^ by glial cells. MPP^+^ has high affinity to DA transporter, and thus preferentially enters dopaminergic neurons [14].

MPP^+^ could accumulate in mitochondria and specifically inhibit the activity of complex I in respiratory transport chain, and result in increased generation of ROS, mitochondrial damage and activation of pro-apoptotic pathways [15]. Available evidence suggests that ROS are served as a key mediator responsible for MPP^+^ induced oxidative damage in DA neuronal cells. ROS can attack essential cell components to trigger oxidation of lipid, DNA, or enzymes, thereby altering several signaling pathways that ultimately promote cell death via apoptosis or necrosis [1,5]. Oxidative stress and mitochondrial dysfunction are also demonstrated in PD patients, and are suggested to be involved in both the initiation and progression of PD [16,17].

In the present study, we investigated the effects of GSRd on MPP^+^ injured SH-SY5Y cells. Our data showed that GSRd increased SH-SY5Y cells survival against MPP^+^ insult. Pre-treatment with GSRd inhibits intracellular ROS formation, reduces the level of MDA, and maintains cellular antioxidant activity (SOD and GPX). So, we proposed that this anti-oxidative effect of GSRd may be partly related to its capability of recruiting the endogenous anti-oxidative system, though the possibility of direct elimination of ROS by GSRd could not be ruled out. GSRd still showed protective effect when MPP^+^ was at a relatively high concentration, which suggested that GSRd may have a direct protection to MPP^+^-induced cytotoxicity.

The protective effect of GSRd was also evidenced by the preservation of mitochondrial function. MMP reflects the function of the electron transport chain and can indicate cell viability. In the present study, we used the cationic fluorescent dye Rh 123, which is sequestered in mitochondria based on the highly negative MMP and released upon mitochondrial depolarization. We found that GSRd could maintain MMP and ATP levels after MPP^+^ treatment. Moreover, we showed that GSRd could restore the activity of respiratory complex I, suggesting that GSRd may play a direct role in preserving complex I and eventually preventing ROS production.

Bax and Bcl-2, members of the pro-apoptotic Bcl-2 family, are important in mitochondria dependent apoptosis. In PD, activation of Bax causes cell death by increasing mitochondrial membrane permeability, eliciting cytochrome *c* release and activating caspase cascade [18]. By contrast, Bcl-2 preserves mitochondrial membrane integrity. So Bax/Bcl-2 ratio has been reported to be an indicator of apoptosis in various disease models [19]. Our findings that GSRd (1 and 10 μM) reduces Bax expression and Bax/Bcl-2 ratio, further support the protective role of GSRd against the toxicity of MPP^+^ in SH-SY5Y cells.

Several kinase-signaling pathways have been shown with great importance in the maintenance of neuronal survival during the pathogenesis of several neurodegenerative diseases, including PD [20,21]. Among them, dysfunction of Akt has been observed in DA neurons of PD patients [22], and adeno-associated virus (AAV) mediated transduction of Akt/Rheb to SN of C57Bl/6 mice could induce re-growth of axons of DA neurons that were previously destroyed by 6-hydroxydopamine [23]. In our previous study, GSRd was shown to inhibit the activity of glycogen synthase kinase-3β (GSK-3β) and enhance the activity of Akt in rats subjected to focal cerebral ischemia [13]. So here we investigated the effect of GSRd on Akt expression in MPP^+^ treated SH-SY5Ycells. Our results showed that Akt activity was decreased after MPP^+^ insult, and GSRd treatment attenuated the down-regulation of P-Akt caused by MPP^+^. Moreover, we found that the protective effect of GSRd was partially blocked by LY294002, an Akt inhibitor. This result suggested that GSRd may also exert its protective effect via the PI3K/Akt survival signaling pathway.

Finally, we preliminarily verified the protective role of GSRd to MPTP damage *in vivo.* Based on our previous investigations, we chose a dose of 10 mg/kg of GSRd [8,12]. Though the optimal dose and potential mechanism are obscure, we found that GSRd partially alleviate the loss of DA neurons caused by MPTP treatment. GSRd is permeable to blood–brain barrier and cell membrane because of its high lipid solubility and steroid-like structure, which may underlie its protective effect on neuronal cells *in vivo* [9,13]. This not only supported our data obtained *in vitro*, but also provided clues for further research on the potential protection roles of GSRd in PD*.*

Some other monomer compounds or water extract of *Panax* *ginseng* are also reported to be protective to MPP^+^ or MPTP-induced toxicity [24,25,26]. In these studies, water soluble ginsenoside Rg1 or Rb1 protects SH-SY5Ycells from MPP^+^ by reducing ROS production, decreasing Bax and caspase-3 expression, alleviating cytochrome *c* leakage and improving mitochondrial function. The formulae of GSRd, Rg1and Rb1 share a common 17-β-estradiol-like structure [27,28]. This may partly explain the common neuroprotective effects of different ginsenosides, but the initial target and mechanism of each are still obscure and further investigation is needed.

## 4. Materials and Methods

### 4.1. Materials

GSRd was obtained from Tai-He Biopharmaceutical (Guangzhou, China). MPP^+^ iodile, MPTP, α-tocopherol and LY294002 were purchased from Sigma-Aldrich (St. Louis, MO, USA). Hoechst 33342, rhodamine 123 (Rh123), 2,7-dichlorofluorescein diacetate (DCFH-DA) were purchased from Beyotime Institute of Biotechnology (Shanghai, China). The commercial kits for detection of lipid peroxidation MDA, SOD, GPX, and ATP bioluminescence were purchased from Nanjing Jiancheng Bioengineering Institute (Nanjing, China). The commercial kit for mitochondrial respiration complex I activity detection was purchased from Genmed Scientifics Corp (Shanghai, China). Antibodies against TH, Bax and Bcl-2 were purchased from Abcam (Cambridge, UK), and antibodies against Akt and P- Akt were purchased from Cell Signaling (Carlsbad, CA, USA). Cy2 conjugated anti-rabbit IgG were purchased from Vector laboratory (Burlingame, CA, USA).

### 4.2. Cell Culture and MPP^+^ Injury

SH-SY5Y cells (Shanghai Institute of Biochemistry and Cell Biology, Shanghai, China) were maintained as previously described [29]. To produce experimental PD model *in vitro*, 150 μM MPP^+^ was added to the cells at a density of 1 × 10^5^/cm^2^ for 72 h based on reports and our pre-test [23]. SH-SY5Y cells were pretreated with different concentrations (1, 10 and 50 μM) of GSRd for 2 h. Then, the medium was removed and cells were treated with 150 μM MPP^+^ in the presence of GSRd for 72 h. The solvent (10% 1,3-propanediol) for dissolving GSRd was served as the vehicle. LY294002 (5 μM) was added 12 h before GSRd. To perform competitive assay, SH-SY5Y cells were treated with different concentrations of MPP^+^ (30, 75, 150, 300 and 600 μM) in combination with constant amount of GSRd (10 μM) or α-tocopherol (10 μM) as the positive control.

### 4.3. MTT and LDH Assays

MTT assay was used to assess cell survival described before [30]. The cell viabilities were represented as the percentages relative to the control. The cell damage was determined based on the release of LDH into the culture supernatant according to the manufacturer’s instructions. The release of LDH was represented as the folds of normal.

### 4.4. Hoechst 33342/PI Double Staining

Hoechst 33342/PI double staining was performed as previously described [29]. As a nucleic acid staining, Hoechst 33342 (bisbenzimide) was utilized to stain nuclei of normal and apoptotic cells. In contrast, PI was impermeable to cells with intact plasma membrane and was used to visualize dead cells. After MPP^+^ treatment, cells were incubated with PI (1 mM) and Hoechst 33342 (5 mM) in the dark at 37 °C for 30 min. For quantification of dead cells, the numbers of PI^+^ cells plus Hoechst 33342^+^ cells with condensed nuclei were counted in a visual field at 200× and at least five different visual fields were included in each group. The percentages of surviving cells to total cells were calculated. Five independent experiments were performed.

### 4.5. ROS, MDA and Antioxidant Enzyme Activity Detection

Levels of ROS and lipid peroxidation MDA, and activities of SOD and GPX were measured as described before [29] using commercial kits. Five independent experiments were performed for each assay. The data were normalized to that of normal.

### 4.6. Mitochondrial Function and ATP Level Examination

MMP measurement was performed and analyzed as described before [29]. ATP bioluminescence was determined following the manufacturer’s instructions. To measure respiratory chain complex I activity, mitochondria were collected by Percoll density gradient centrifugation. Before measurement, mitochondria membrane was disrupted and enzymes were exposed by three freeze–thaw cycles. Activities of complex I (NADH dehydrogenase) was measured following the manufacturer’s instructions, and was indicated by the consumption of NADH per minute per unit mass. Five independent experiments were performed for each measurement. The data were normalized to that of normal.

### 4.7. Western Blot Analysis

Western blot analysis of Bax, Bcl-2, Akt and P-Akt was performed as previously described [31]. Proteins of SH-SY5Y cells were extracted using a protein extraction kit (Beyotime, Shanghai, China), according to the manufacturer’s instructions. After electrophoresed on 10% SDS-PAGE, proteins in the gels were transferred onto nitrocellulose membranes, which were incubated with the primary antibody (anti-Bax at 1:1000, anti-Bcl-2 at 1:500, anti-Akt at 1:2000, anti-P-Akt at 1:500 and anti-β-actin at 1:3000). After washed with TBST (0.1% Tween 20 in TBS), the membranes were incubated with horseradish peroxidase-conjugated secondary antibody (Amersham, NJ, USA) for 1 h at room temperature and developed using an enhanced chemiluminescence Western blotting detection kit (Pierce Biotechnology, Rockford, IL, USA). Five independent experiments were performed. The data were normalized to β-actin expression and further normalized to the control.

### 4.8. Animals and MPTP-Induced Dopaminergic Lesion

Adult male C57BL/6J mice (22–25 g) obtained from the Center of Experimental Animal in the Fourth Military Medical University were housed as previously described [31]. Mice were randomly divided into 4 groups, each containing 5 mice. To induce DA neurons lesion, mice were intraperitoneally injected with MPTP (30 mg/kg) once in a day for 5 consecutive days. In experimental group, mice were intraperitoneally injected with GSRd (10 mg/kg) 2 h prior to every MPTP injection once in a day. Then GSRd treatment continued another 16 consecutive days with a total of 21 days. The vehicle group was injected with 10% 1,3-propanediol. Animals were sacrificed 1 day after the final injection of GSRd. The experimental procedure in this study was approved by the Institutional Animal Care and Use Committee at the Fourth Military Medical University and all applicable guidelines for the care and use of animals were followed.

### 4.9. Immunostaining Assays

Immunostaining assays of TH positive DA neurons were performed as previously described [31]. Coronal floating sections through SN with 20 μm thick were incubated with rabbit anti-TH (1:500) in PBS at 4 °C overnight, and then incubated with Cy2 conjugated anti-rabbit IgG (1:300) for 2 h at room temperature. Immunostaining signals were observed under a Leica DMIRB microscope (Leica, Wetzlar, Germany).

### 4.10. Statistical Analysis

Data were expressed as mean ± SD. Statistical analysis was performed using the SPSS 19.0 software. Differences were determined with one-way ANOVA followed by least significant difference (LSD) *t* test. Values were considered to be significant when *p* < 0.05.

## 5. Conclusions

In conclusion, our research provides the first evidence that GSRd exerts protective effects on MPP^+^ and MPTP induced experimental PD models, which may be ascribed to its antioxidant effects and mitochondrial function preservation.

## References

[1] Onyango I.G. (2008). Mitochondrial dysfunction and oxidative stress in parkinson’s disease. Neurochem. Res..

[2] Perfeito R., Cunha-Oliveira T., Rego A.C. (2012). Revisiting oxidative stress and mitochondrial dysfunction in the pathogenesis of parkinson disease—Resemblance to the effect of amphetamine drugs of abuse. Free Radic. Biol. Med..

[3] Hauser D.N., Hastings T.G. (2013). Mitochondrial dysfunction and oxidative stress in parkinson’s disease and monogenic parkinsonism. Neurobiol. Dis..

[4] Macchi B., Paola D.R., Marino-Merlo F., Felice M.R., Cuzzocrea S., Mastino A. (2015). Inflammatory and cell death pathways in brain and peripheral blood in parkinson’s disease. CNS Neurol. Disord. Drug Targets.

[5] Yan M.H., Wang X., Zhu X. (2013). Mitochondrial defects and oxidative stress in alzheimer disease and parkinson disease. Free Radic. Biol. Med..

[6] Ye R., Han J., Kong X., Zhao L., Cao R., Rao Z., Zhao G. (2008). Protective effects of ginsenoside Rd on PC12 cells against hydrogen peroxide. Biol. Pharm. Bull..

[7] Ye R., Li N., Han J., Kong X., Cao R., Rao Z., Zhao G. (2009). Neuroprotective effects of ginsenoside Rd against oxygen-glucose deprivation in cultured hippocampal neurons. Neurosci. Res..

[8] Ye R., Kong X., Yang Q., Zhang Y., Han J., Li P., Xiong L., Zhao G. (2011). Ginsenoside Rd in experimental stroke: Superior neuroprotective efficacy with a wide therapeutic window. Neurochem. Int..

[9] Ye R., Yang Q., Kong X., Han J., Zhang X., Zhang Y., Li P., Liu J., Shi M., Xiong L. (2011). Ginsenoside Rd attenuates early oxidative damage and sequential inflammatory response after transient focal ischemia in rats. Neurochem. Int..

[10] Ye R., Zhang X., Kong X., Han J., Yang Q., Zhang Y., Chen Y., Li P., Liu J., Shi M. (2011). Ginsenoside Rd attenuates mitochondrial dysfunction and sequential apoptosis after transient focal ischemia. Neuroscience.

[11] Liu X., Xia J., Wang L., Song Y., Yang J., Yan Y., Ren H., Zhao G. (2009). Efficacy and safety of ginsenoside-Rd for acute ischaemic stroke: A randomized, double-blind, placebo-controlled, phase II multicenter trial. Eur. J. Neurol..

[12] Gonzalez-Polo R.A., Soler G., Rodriguezmartin A., Moran J.M., Fuentes J.M. (2004). Protection against MPP^+^ neurotoxicity in cerebellar granule cells by antioxidants. Cell Biol. Int..

[13] Zhang X., Shi M., Ye R., Wang W., Liu X., Zhang G., Han J., Zhang Y., Wang B., Zhao J. (2014). Ginsenoside Rd attenuates tau protein phosphorylation via the PI3K/AKT/GSK-3β pathway after transient forebrain ischemia. Neurochem. Res..

[14] Zhai A., Zhu X., Wang X., Chen R., Wang H. (2013). Secalonic acid a protects dopaminergic neurons from 1-methyl-4-phenylpyridinium (MPP^+^)-induced cell death via the mitochondrial apoptotic pathway. Eur. J. Pharmacol..

[15] Perier C., Bove J., Wu D.C., Dehay B., Choi D.K., Jackson-Lewis V., Rathke-Hartlieb S., Bouillet P., Strasser A., Schulz J.B. (2007). Two molecular pathways initiate mitochondria-dependent dopaminergic neurodegeneration in experimental parkinson’s disease. Proc. Natl. Acad. Sci. USA.

[16] Keeney P.M., Xie J., Capaldi R.A., Bennett J.P. (2006). Parkinson’s disease brain mitochondrial complex I has oxidatively damaged subunits and is functionally impaired and misassembled. J. Neurosci..

[17] Isobe C., Abe T., Terayama Y. (2010). Levels of reduced and oxidized coenzymeQ-10 and 8-hydroxy-2′-deoxyguanosine in the cerebrospinal fluid of patients with living parkinson’s disease demonstrate that mitochondrial oxidative damage and/or oxidative DNA damage contributes to the neurodegenerative process. Neurosci. Lett..

[18] Perier C., Tieu K., Guegan C., Caspersen C., Jackson-Lewis V., Carelli V., Martinuzzi A., Hirano M., Przedborski S., Vila M. (2005). Complex I deficiency primes bax-dependent neuronal apoptosis through mitochondrial oxidative damage. Proc. Natl. Acad. Sci. USA.

[19] Erfani S., Khaksari M., Oryan S., Shamsaei N., Aboutaleb N., Nikbakht F. (2015). Nampt/PBEF/visfatin exerts neuroprotective effects against ischemia/reperfusion injury via modulation of Bax/Bcl-2 ratio and prevention of caspase-3 activation. J. Mol. Neurosci..

[20] Wang G., Pan J., Chen S.D. (2012). Kinases and kinase signaling pathways: Potential therapeutic targets in parkinson’s disease. Prog. Neurobiol..

[21] Wada A., Yokoo H., Yanagita T., Kobayashi H. (2005). Lithium: Potential therapeutics against acute brain injuries and chronic neurodegenerative diseases. J. Pharmacol. Sci..

[22] Timmons S., Coakley M.F., Moloney A.M., O’Neill C. (2009). Akt signal transduction dysfunction in parkinson’s disease. Neurosci. Lett..

[23] Kim S.R., Chen X., Oo T.F., Kareva T., Yarygina O., Wang C., During M., Kholodilov N., Burke R.E. (2011). Dopaminergic pathway reconstruction by Akt/Rheb-induced axon regeneration. Ann. Neurol..

[24] Hu S., Han R., Mak S., Han Y. (2011). Protection against 1-methyl-4-phenylpyridinium ion (MPP^+^)-induced apoptosis by water extract of ginseng (*Panax ginseng* C.A. Meyer) in SH-SY5Y cells. J. Ethnopharmacol..

[25] Chen X.C., Zhou Y.C., Chen Y., Zhu Y.G., Fang F., Chen L.M. (2005). Ginsenoside Rg1 reduces MPTP-induced substantia nigra neuron loss by suppressing oxidative stress. Acta Pharmacol. Sin..

[26] Radad K., Gille G., Moldzio R., Saito H., Ishige K., Rausch W.D. (2004). Ginsenosides Rb1 and Rg1 effects on survival and neurite growth of MPP^+^-affected mesencephalic dopaminergic cells. J. Neural Trans..

[27] Shi C., Zheng D.D., Fang L., Wu F., Kwong W.H., Xu J. (2012). Ginsenoside Rg1 promotes nonamyloidgenic cleavage of APP via estrogen receptor signaling to MAPK/ERK and PI3K/AKT. Biochim. Biophys. Acta.

[28] Ni N., Liu Q., Ren H., Wu D., Luo C., Li P., Wan J.B., Su H. (2014). Ginsenoside rb1 protects rat neural progenitor cells against oxidative injury. Molecules.

[29] Zhao J., Bai Y., Zhang C., Zhang X., Zhang Y.X., Chen J., Xiong L., Shi M., Zhao G. (2014). Cinepazide maleate protects pc12 cells against oxygen-glucose deprivation-induced injury. Neurol. Sci..

[30] Cen J., Liu L., He L., Liu M., Wang C.J., Ji B.S. (2012). N^1^-(quinolin-2-ylmethyl)butane-1,4-diamine, a polyamine analogue, attenuated injury in *in vitro* and *in vivo* models of cerebral ischemia. Int. J. Dev. Neurosci..

[31] Shi M., Du F., Liu Y., Li L., Cai J., Zhang G.F., Xu X.F., Lin T., Cheng H.R., Liu X.D. (2013). Glial cell-expressed mechanosensitive channel TRPV4 mediates infrasound-induced neuronal impairment. Acta Neuropathol..

